# Disruption of biological rhythms as a core problem and therapeutic target in mood disorders: the emerging concept of 'rhythm regulators'

**DOI:** 10.1186/1744-859X-9-3

**Published:** 2010-01-13

**Authors:** Konstantinos N Fountoulakis

**Affiliations:** 1 Third Department of Psychiatry, Aristotle University of Thessaloniki, Thessaloniki, Greece

## Abstract

Biological rhythms have always been considered to be disrupted in depression, with the predominant theory being that of hyperarousal. However, recent data suggest that it might be more appropriate to suggest that depressed patients are incapable of achieving and maintaining the particular level of internal homeostasis which permits them to function smoothly, to lower the level of arousal during sleep sufficiently so that quality of sleep is good, and to increase this level enough during the day so the person can function properly. Therefore, the transition from one state to another is somewhat problematic, delayed, incomplete and desynchronised. Thus, agents with a 'rhythm stabilising' effect could be beneficial in the treatment of mood disorders. Such an agent should have a beneficial effect on restoring and stabilising the rhythm of a physiological function while not pushing it towards a specific pole, or inducing the opposite pole; it should also allow response to internal and environmental stimuli and zeitgebers, and restore synchronisation of the various body rhythms while not inducing or worsening desynchronisation. Agomelatine could represent the first of a new class of 'rhythm stabilising antidepressants', but further research is necessary to support this theory.

## Introduction

Biological rhythms have always been considered to be disrupted in depression [1]. In the seminal paper by Akiskal and McKinney [2] it has been concluded that depression is related to hyperarousal, because depressive patients have lower threshold for awakening, rapid eye movement (REM) sleep disorders and loss of delta waves related sleep, which is the deepest stage of non-REM sleep. By 'hyperarousal', it is denoted that depressed patients manifest both higher reactivity to environmental stimuli and sympathetic nervous system (SNS) hyperactivity. According to this approach, sleep disorders are considered in the frame of higher vigilance [3]. Some authors reject this theory and suggest that only somatisation can involve altered central nervous system (CNS) processing (hyperarousal) of somatic stimuli [4]. Theoretically, a disturbance of biological rhythms could be a core feature in the etiopathogenesis of depression [5-7].

However, from a clinical point of view depression is not a uniform clinical entity. Even for unipolar depression it is not known whether it is a single disorder or a spectrum of different disorders with overlapping clinical manifestations. Clinically, depressed patients do not manifest sleep disorders alone (difficulty in initiating or sustaining sleep or low sleep quality). The same patients, when awake, manifest concentration difficulties, reduced psychomotor activity, fatigue, somnolence and so on, and these symptoms could be independent from the sleep they experienced the previous night (that is, they are not always consequences of poor sleeping).

Melancholic patients in particular typically manifest sleep disorders during the night (insomnia) and fatigue, concentration difficulties and anhaedonia with a lack of prominent anxiety during the daytime. These could be suggestive of a reduction of the arousal level variation span. According to this theory, the arousal level is not sufficiently reduced during the night so as to lead to normal sleep, while during the daytime it is not sufficiently increased so as to lead to normal functioning. On the contrary, atypical patients manifest hypersomnia, higher levels of anxiety and personality disorders as well as increased reactivity towards the environment. These are suggestive of an increased variation range of the arousal level, which during the night could be too low, leading to a prolonged but low quality sleep, while during the daytime the level may be too high, thus leading to anxiety and hyperactivity.

So although the hyperarousal theory for depression is generally accepted and mainly derives from the consistent finding of a lower threshold for awakening, rapid eye movement (REM) sleep disturbances and loss of delta-activity-related sleep, the data in the literature suggest that the generalised term 'hyperarousal' could be misleading in the case of depression. Depression is characterised by a disruption of the span of variation of the activity of several brain region functions and a desynchronisation of the sequence of activation/deactivation of various neural circuits, as a response to external stimuli or to states of the organism. This results in the compromise of many functions, and may constitute the cause for the formation of symptomatology. This could be true both at the level of specific brain regions and at the level of coordination between different brain structures, and it can be state or syndrome dependent [8-10].

Additionally, anxiety is generally considered to be a sign of an increased level of alertness, however it might be wrong to identify these two concepts as related. Anxiety and nervousness are the consequences of poor sleep or prolonged insomnia even in normal subjects, but are not present under standard and normal conditions; at those hours of the day the circadian cycle indices have the greatest difference from the respective values during sleep. That is, anxiety is not related to those periods of the day when alertness is at its highest.

Taking all the above into consideration, it might be more appropriate to suggest that depressed patients are incapable of achieving and maintaining the particular level of internal homeostasis that permits the person to function smoothly, to lower enough the level of arousal during sleep so that quality of sleep is good, and to increase this level enough during the day so that the person can function properly. The same seems to hold true not only for circadian but also for ultradian variations. Therefore the transition from one state to another is somewhat problematic, delayed, incomplete and desynchronised. This is in accord with the suggestion of Bruder *et al. *[11] that a fused arousal/attentional system involving temporoparietal and possibly frontal regions is dysfunctional in depression, and the suggestion of Shagas *et al. *[12,13] that depressed patients may be underactivated at rest but over-reactive after stimulation.

This disregulation of rhythms could be either a core etiopathogenetic factor or a severe manifestation of depression, and therefore needs specific evaluation and treatment [14,15]. Treating with a hypnotic or sedative is not appropriate; on the contrary, there are reports in the literature that such a treatment might worsen the long term outcome of these patients, but this could be mainly because of the addiction patients develop.

Thus agents with a 'rhythm stabilising' effect could be beneficial in the treatment of mood disorders. The term 'rhythm stabiliser' could be defined in a similar way to how the term 'mood stabilizer' is perceived today [16-18], that is 'a compound that has been shown to be efficacious in recurrence prevention of manic and depressive symptoms, but not necessarily in the treatment of the acute manic or depressive phase, although it would be an advantage if it were' [16]. A similar more realistic definition suggests that the term 'mood stabiliser' describes an agent that ideally 'would prevent relapse to either pole of the illness' [16] without causing a negative effect on other phases of the illness [19]. Following this path, the criteria that could define a 'rhythm stabiliser' can be specific and operational but of course only further research can provide answers.

Such an agent should have a beneficial effect on the rhythm of a physiological function (restoring and stabilising it) and not pushing it towards a specific pole (as, for example, hypnotics do). This restoration should include the whole pattern of the rhythm of the function under consideration (period/frequency and amplitude) and its relationship to other body functions. Some rhythms have a very complex pattern and periodicity and the restoration should be as close to normality as possible, but at the same time the fixation to normality should not be too rigid and should allow response to both internal and environmental stimuli and zeitgebers and space for adaptation to change if needed. The question of whether an effect on a single rhythm would be sufficient to warrant the characterisation of a 'rhythm stabilizer' or a broader effect on multiple physiological functions is necessary, cannot be addressed sufficiently today. A broad definition will include even compounds acting on a single function. The most important property of a 'stabiliser' would be to not induce the opposite pole in the physiological function it is supposed to restore or in any other function, and further, to restore synchronisation of the various body rhythms and not induce or worsen dysynchronisation.

A final question concerns the source of data suggesting the agent has 'stabilised' the rhythm. They could come from direct clinical observations and interviews with the subject, or from laboratory investigation. This is also a very difficult issue to answer because there might be at least some functions (for example, sleep, daily energy and fatigue) that can be accessed through clinical observation and interview (at least to a fair degree), however the vast majority of functions would need sophisticated laboratory assessment, which is not available in most clinical settings. An 'either/or' phrase might be the best compromise for the time being.

A summary of proposed criteria for further study concerning the definition and the properties a 'rhythm stabiliser' should fulfil is presented in Table 1.

**Table 1:** Criteria for a rhythm stabiliser

Criteria	Description
1	Efficacy in restoring and stabilising at least one physiological function (not restricted to imposing a specific effect like hypnotics do)

A	Restores the internal properties of the rhythm, that is period/frequency and amplitude

B	Corrects the internal pattern of complex rhythms

2	By correcting the abnormal overexpression towards the one pole, the agent:

A	Does not induce the opposite pole of the specific function under treatment

B	Does not induce the opposite pole of any other physiological function which has a rhythm

C	Does not induce desynchronisation of rhythms by correcting a specific rhythm or rhythms

3	It synchronises different functions:

A	Between them

B	With the environment

4	Restoration is flexible; permits adjustment to change

A	Permits normal response to zeitgebers

B	Permits normal response stimuli (internal or external)

5	The effect is evident either clinically or after laboratory investigation

In the frame of the above, the development of agomelatine could represent a new approach to the treatment of depression, since it could be the first compound that fulfils the criteria for a 'rhythm stabilising antidepressant'. Unlike other antidepressants, agomelatine has a novel neurochemical mechanism of action since it is an MT1 and MT2 melatonergic receptor agonist and an antagonist at the 5-HT2C receptor. By interacting with these receptor subtypes it causes the restoration of circadian rhythms [20]. Randomised controlled trials suggest that it leads to a global and rapid amelioration of depression by relieving the core symptoms of depression, including depressed mood, anxiety symptoms, and those that frequently remain untouched by other antidepressants (for example, sleep/wake disturbances), while important functions such as sexual function remain intact. At the same time, remission rates are reported to be satisfactorily high [21].
